# Oncology Nurse Practitioner Web Education Resource(ONc-PoWER): An Evaluation of a Web-Enhanced Education Resource for Nurse Practitioners Who Are New to Cancer Care

**Published:** 2018-01-01

**Authors:** Rosemary L. Hoffmann, Sara Klein, Mary Connolly, Margaret Quinn Rosenzweig

**Affiliations:** University of Pittsburgh, School of Nursing, Pittsburgh, Pennsylvania

## Abstract

The nurse practitioner (NP) new to an advanced role in a specialty practice may find the orientation period challenging while expanding his or her professional knowledge base. The Oncology Nurse Practitioner Web Education Resource (ONc-PoWER), a web-enhanced continuing education program, provides training for both the oncology NP (ONP) new to cancer care (i.e., within the first year) and his or her on-site mentor. The Oncology Nurse Practitioner Web Education Resource promotes essential didactic and skills development via five modules. A total of 103 dyads (i.e., a new ONP and his or her mentor) were recruited from 27 states to participate in ONc-PoWER, and 79 dyads completed surveys to evaluate the program. Data were collected between April 2012 and October 2016. We observed statistical significance (*p* = .000) between pre- and post-program self-assessment of cancer care knowledge and confidence in delivering cancer care among ONPs. Moreover, mentors rated the program favorably. Mentor agreement concerning whether or not the ONPs were able to successfully perform 30 core clinical skills ranged from a low of 93% to a high of 100%. Furthermore, mean scores of the course satisfaction survey demonstrate that each ONc-PoWER objective either met or exceeded learner expectations.

Professional nursing education is a continuous process that begins with entry into a basic nursing program and continues throughout the nursing career, which often encompasses entry into advanced practice nursing. In 2009, both the American Association of Colleges of Nursing Board of Directors and the Association of American Medical Colleges Council of Deans articulated a vision for a health professional education continuum. According to this vision, the continuum ranges from entry into practice to retirement and "values, exemplifies, and assesses lifelong learning skills; emphasizes interprofessional and team-based education and practice; employs tested outcomes-based continuing education methods; and links health professional education and delivery of care within the workplace" (American Association of Colleges of Nursing, 2010). Lifelong learning of this sort prepares graduates with skills that support ongoing practice and learning through continuing educational methods and self-learning opportunities. In particular, one of the methods is the use of technology to deliver evidence-based information and assess changes in practice (American Association of Colleges of Nursing, 2010).

Lifelong learning is a challenge for nurses across the spectrum of care in general and disease-focused practice (American Association of Colleges of Nursing, 2010). For example, nurses report time and financial constraints, along with staff shortages, limiting their ability to participate in continuing educational opportunities (Bahn, 2006). Therefore, opportunities to further expand the knowledge base of professional nurses vis-à-vis (1) specialized patient care practice content; (2) professional development; and (3) patient care delivery to maintain professional practice commensurate with the needs of these working professionals are necessary to effectively promote lifelong learning in nursing.

Research examining the effectiveness of nurse practitioners (Bishop, 2009; Carper & Haas, 2006; Hoffman, Tasota, Zullo, Scharfenberg, & Donahoe, 2005; Kinney, Hawkins, & Hudmon, 1997; Levy, Gagnet & Stewart, 2013; McCabe et al., 2013; Young, 2005) provides strong evidence of the cost effectiveness, patient satisfaction, and quality care outcomes in primary care, acute care, and specialty practices that are associated with the nurse practitioner (NP) role. Specifically, the autonomous role of the NP in specialty and acute care contributes to positive patient outcomes (Newhouse et al., 2011), which include, but are not limited to fewer hospital admissions (Collins et al., 2014; Jennings, Clifford, Fox, O’Connell, & Gardner, 2015; Oliver, Pennington, Revelle, & Rantz, 2014), decreased length of stay (Collins et al., 2014; Jennings et al., 2015; Oliver et al., 2014), fewer readmission rates and emergency care visits (Laurent et al., 2005), and reduced health-care costs (Oliver et al., 2014).

One important care setting that has embraced the use of NPs is cancer care (Dyar, Lesperance, Shannon, Sloan, & Colon-Otero, 2012; Hinkel et al., 2010; Kelly & Agius, 2006; Kinney et al., 1997; Leonard, & Grossman, 2010). In cancer care, the oncology NP (ONP) (1) contributes specialty knowledge through the assessment of patients and families across the cancer continuum; (2) provides assessment and treatment for common side effects of cancer and cancer treatment; (3) assists patients by educating them about treatment-specific side effects; and (4) supports patients and families with decision-making concerning the coordination of survivorship and/or end-of-life care (Rosenzweig, Giblin, Morse, Sheehy, & Sommer, 2012).

Although ONPs can offer value to the care provided for cancer patients, the opportunities for ONPs to contribute fully to this care may not be realized for several reasons, which include (1) the ONP’s lack of expertise about a given cancer type; (2) the physician’s lack of familiarity with the potential of the ONP role; and (3) poor onboarding or orientation to the role of the autonomous cancer care provider (i.e., ONP). For example, according to Rosenzweig et al. (2012), NPs who are new to cancer care can demonstrate their familiarity with the generic NP role (history and physical, formation of a differential diagnosis); nonetheless, these NPs often lack familiarity with the specific knowledge and tasks related to the provision of cancer care. As a result of poor orientation, a new ONP may assume the more comfortable and familiar role of the "staff nurse" rather than that of the advanced practice nurse, which ultimately limits the potential of the ONP. These issues clearly exemplify the need for not only an improved ONP orientation (e.g., one that is creative and clinician-mentored [i.e., by a physician, NP, or PA]), but also some sort of standardized, entry-level cancer care education for both ONPs and other hospital personnel (e.g., on-site mentors).

The continuing education program, the Oncology Nurse Practitioner Web Education Resource (ONc-PoWER), was developed to provide educational content for both ONPs who are new to cancer care (i.e., within their first year in the role) and information for their respective mentors. The ONc-PoWER curriculum was funded through the National Cancer Institute (R25 1R25CA148050-01A1), facilitating the essential introductory didactic and skills development that is provided by way of five modules (see Table 1): (1) The New Patient; (2) Patient Presentation; (3) Continuum of Care; (4) Palliative and Hospice Care; and (5) Self-Care and Professional Development.

**Table 1:**
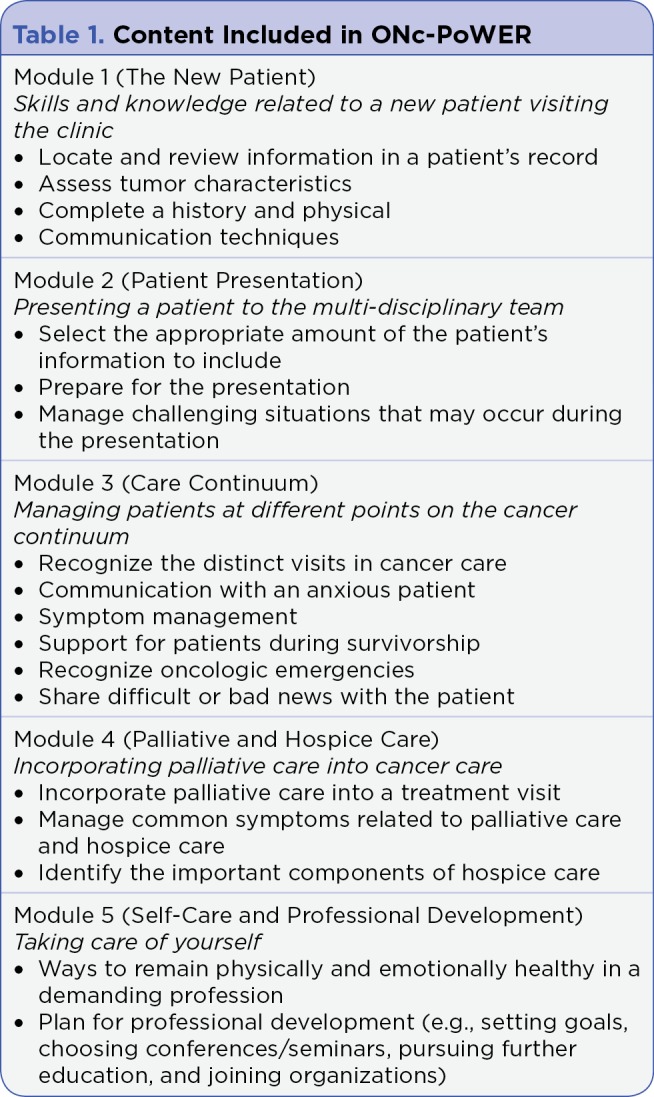
Content Included in ONc-PoWER

The framework for the development and dissemination of ONc-PoWER has been described in a prior article (Hoffmann, Klein, & Rosenzweig, 2016). The five modules of ONc-PoWER introduce learners to an avatar named Gina (see Figure 1), who represents a recent NP graduate and new ONP who faces everyday challenges and tasks in her health-care context. Through an asynchronous learning format based on (1) adult learning theory; (2) principles of quality online development; and (3) features of serious gaming, Gina has the look and sound of a new ONP who is excited yet nervous about her new position. Throughout the five modules, Gina is assigned an on-site, clinical mentor named Sandra (see Figure 2), another avatar, who provides support, direction, and encouragement. It is through Sandra that ONc-PoWER helps Gina solve the dilemmas of being a new ONP by presenting content germane to navigating her new role.

**Figure 1:**
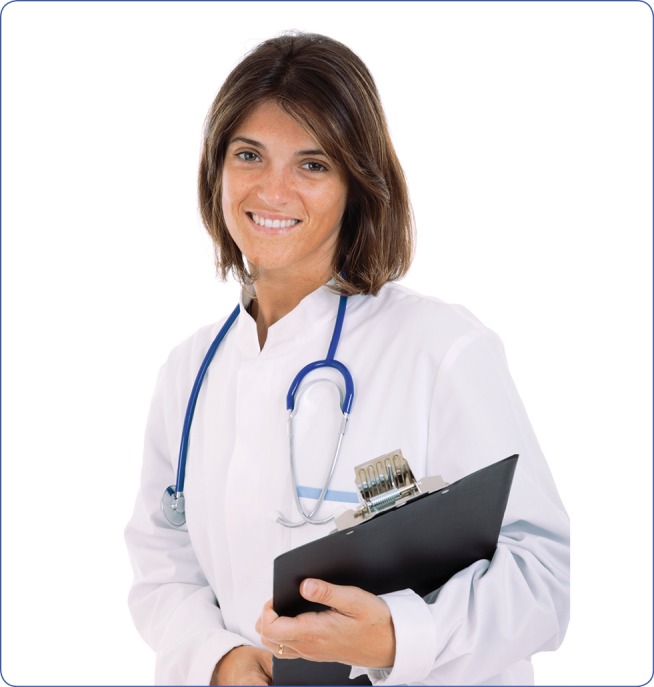
Gina, a new ONP.

**Figure 2:**
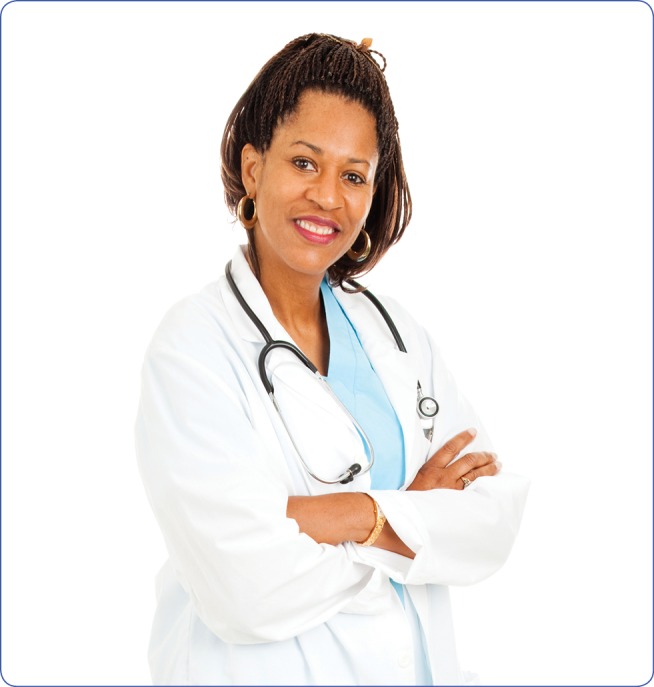
Sandra, an ONP mentor

This paper describes the evaluation of an electronic, continuing education program to facilitate the orientation process for ONPs who are new to cancer care with the support of the on-site mentor. The specific aims were (1) to evaluate the impact of the ONc-PoWER curriculum on the ONP’s cancer knowledge after completion of the five modules; (2) to assess the mentor’s ability to measure the ONP’s application of designated skills of oncology clinical practice as presented in the five ONc-PoWER modules; and (3) to assess the ONP’s and mentor’s evaluation of the ONP’s attainment of curriculum objectives. In addition, this paper evaluates the ONc-PoWER curriculum modules to determine the effectiveness, usefulness, and applicability of this continuing education opportunity for nursing.

## PROCEDURE

Recruiting for enrollment in ONc-PoWER and occurred in the following ways. There were national recruitment efforts through social media, primarily focused on the LinkedIn and Facebook platforms. Recruitment strategies included a table display advertising the program and presentations at national ONP conferences, in addition to written content for nursing trade journal discussions. Referrals and word of mouth from advanced practice providers (APPs) and ONPs were additional recruitment methods.

Interested ONPs and/or their mentors contacted the University of Pittsburgh School of Nursing and provided demographic information. The ONc-PoWER research staff then contacted the interested participant and determined eligibility status, which included a nurse practitioner working in cancer care, first year of oncology APP practice, and an on-site mentor (physician, NP, or PA). If the participant met eligibility status, the ONc-PoWER staff sent a link to register through the University of Pittsburgh CourseWeb site (i.e., BlackBoard platform).

Once the participants began working through the five ONc-PoWER modules, their progress and time to completion were monitored by study staff. The ONc-PoWER modules were designed to be completed over a 4- to 6-month period. If participants were not on track to complete the modules at the 5-month time point, an email was sent to the given participant reminding him or her of the 6-month deadline to finish ONc-PoWER. All ONPs enrolled completed evaluations for each module as well as a final course evaluation. They also completed a pre- and post-knowledge and confidence scale. The mentors completed a final course evaluation.

## MEASURES

To measure the participants’ pre- and post-training knowledge of cancer, an investigator-developed evaluation was designed to measure the knowledge of the new ONP and his or her ability to complete key tasks that are essential to providing safe, quality cancer care. The knowledge and key tasks were determined in accordance with the core competency of the Oncology Nursing Society (ONS) for entry into practice for an NP (ONS, 2007). The ONPs completed the knowledge pre-test prior to accessing the first module. They completed the post-test after the fifth module. The pre- and post-test knowledge evaluations contained 10 multiple-choice item questions (e.g., "What is the first step to gathering information for a new patient history and physical?"). Meanwhile, the mentors were asked to respond "yes" or "no" to 30 questions involving clinical activities (e.g., "The NP identified a list of current clinical trials available to our patient population and he/she is familiar with the process of clinical trial recruitments") that evaluated the ability of their respective ONPs to perform the core clinical tasks presented in each of the modules. There were 11 tasks for The New Patient module, 5 for the Patient Presentation module, 7 for the Continuum of Care module, 4 for the Palliative and Hospice Care module, and 3 for the Self-Care and Professional Development module.

Following the completion of the ONc-PoWER curriculum, both ONPs and mentors were asked to complete a course evaluation. To ensure confidentiality, the evaluation was distributed electronically via the Research Electronic Data Capture (REDCap) tools as detailed by Harris et al. (2009; project-redcap.org). The evaluation consisted of seven items (e.g., "Understand how to prepare for a new patient visit") and the participants responded to each of these items with responses ranked on a five-point Likert scale: 1 = Did not meet objective, 2 = Somewhat met objective, 3 = Met objective, 4 = More than met objective, and 5 = Exceeded objective expectation. In addition, the course evaluation featured two open-ended items: (1) Identify two things that you have learned from this activity; and (2) Can you identify any content that you desired that was not included in the learning activities?

## DATA MANAGEMENT AND EVALUATION

Data were first collected through the University of Pittsburgh CourseWeb (i.e., BlackBoard) gradebook feature, and then exported to the secure REDCap database hosted at the University of Pittsburgh. The research team followed participants enrolled in the curriculum daily. Study progress, with attention to evaluative data, was reviewed weekly by the research team and primary investigator. IBM SPSS Statistics 22 software was used for data management and analysis. Descriptive statistics and a paired sample T-test were used for analysis.

## RESULTS

**Figure 3:**
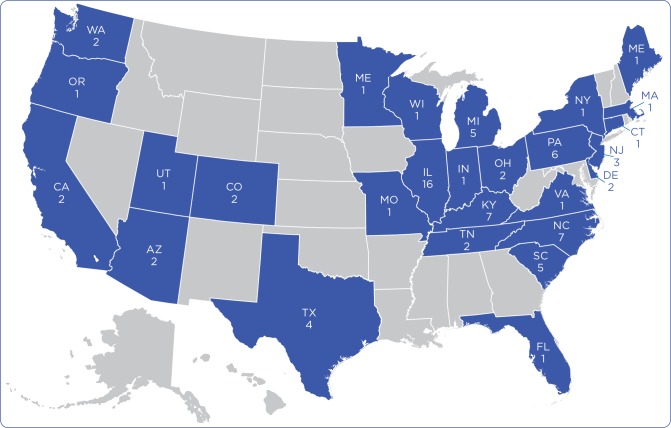
Map showing numbers of ONP and mentor participants and their states of residence.

Data were collected between April 2012 and October 2016. One hundred and three dyads (i.e., new ONP and his or her mentor) were recruited. Seventy-nine ONPs completed (1) the pre- and post-program knowledge and task evaluation; and (2) the end-of-course evaluation. Seventy-nine mentors completed the core clinical skills ONP evaluation. Dyads were recruited from 27 states (see Figure 3). "Lack of time" was the primary reason cited for attrition.

**Demographics**

The demographic data collected reflect the current NP workforce: all but three of the ONPs were female, and 96% were Caucasian. The ONPs resided in 27 states, and 41% had 6 to 10 years of experience as a registered nurse (RN). Most of the ONPs (79%; n = 62) had less than 1 year of experience as NPs, and only one NP had greater than 15 years of experience. The majority of the ONPs were prepared as adult gerontology nurse practitioners (AGNP; n = 38; 48%); 35 (44%) were prepared as family nurse practitioners (FNP), and six (7%) as acute care nurse practitioners (ACNP; n = 6; 7%; Table 2). Sixty-one (77%) of the mentors were NPs (i.e., geriatric, adult, acute care, family, or women’s health). Eight (10%) were MD/DOs, and the remaining mentors (n = 10; 13%) were PAs. Twenty-three (29%) of the mentors had greater than 20 years of experience in cancer care, 17 (21%) had 6 to 10 years of experience, and 16 (20%) had 2 to 5 years of cancer care experience (see Table 3).

**Table 2:**
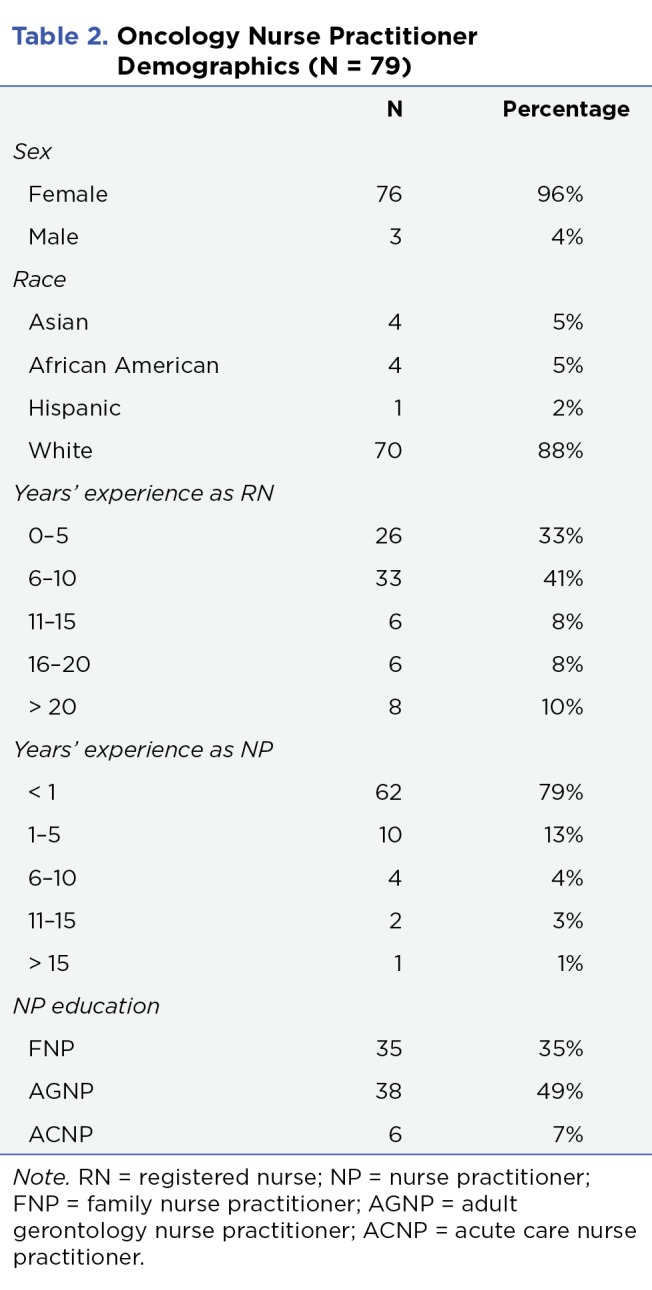
Oncology Nurse Practitioner Demographics (N = 79)

**Table 3:**
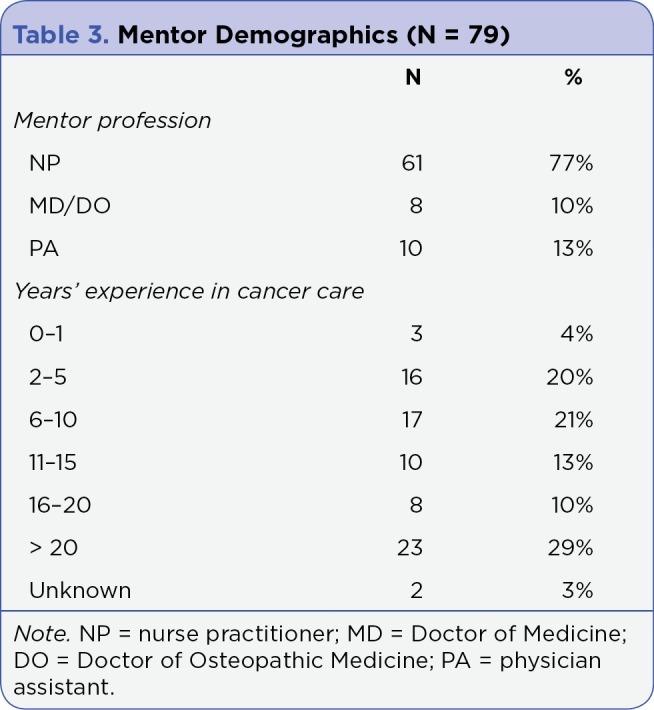
Mentor Demographics (N = 79)

**Aim 1**

The first aim was to assess the impact of the ONc-PoWER curriculum on the ONP’s cancer knowledge after completing the five modules. A paired sample T-test compared matched pre- and post-program scores. The difference between pre- and post-program self-assessment of cancer care knowledge and confidence in delivering cancer care among the ONPs was statistically significant (*p* = .000).

**Aim 2**

The given mentor’s measurement of the ability of his or her ONP to apply the designated skills of oncology clinical practice, as presented in the modules of ONc-PoWER, was also assessed. Seventy-nine of the mentors responded "yes" or "no" to the 30 questions asking whether or not the ONP could perform core clinical skills. Mentor agreement that the new ONPs were able to successfully perform the 30 core clinical skills ranged from a low of 93% to a high of 100%. In addition, the mentors rated the program favorably (see Table 4).

**Table 4:**
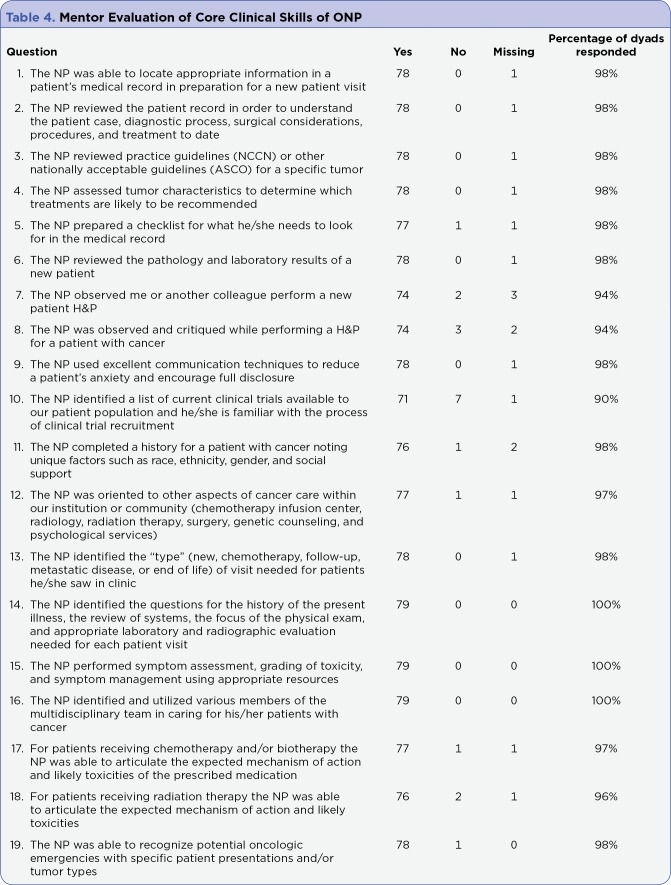
Mentor Evaluation of Core Clinical Skills of ONP

**Table 4 cont:**
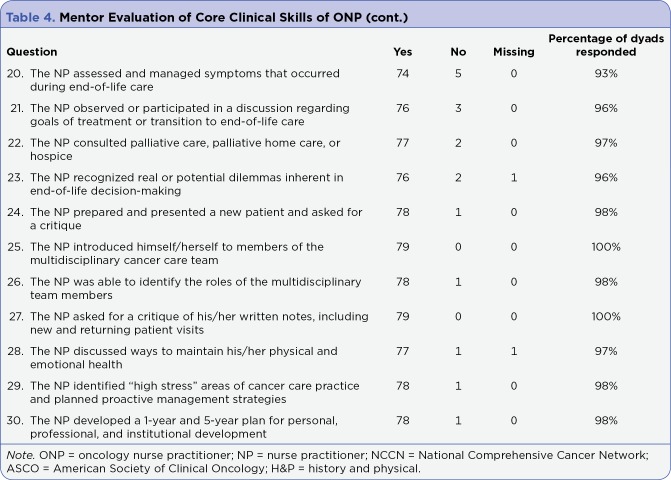
Mentor Evaluation of Core Clinical Skills of ONP (cont.)

**Aim 3**

There was both a qualitative and quantitative evaluation of the ONc-PoWER curriculum. Both the mentors and ONPs rated the ability of the ONPs to perform core clinical practice tasks that reflected the objectives of the ONc-PoWER curriculum. This quantitative measure was obtained from responses on a five-point Likert scale (i.e., 1 = Did not meet objective; 5 = Exceeded objective evaluation). Mean scores from the mentor evaluation of the ONP ranged from a high of 4.01 (i.e., "Understands the similarities and differences of palliative care vs. hospice care and when to incorporate that care into patient visits") to 3.87 (i.e., "Understands how to maintain physical and emotional health"). The learning needs attainment mean scores for the ONPs ranged from a high of 3.78 (i.e., "Understands how to prepare for a new patient visit") to 3.55 (i.e., "Applies the content to practice"). All mean scores reflect that each objective either met or more than met the learner’s expectations (see Table 5).

**Table 5:**
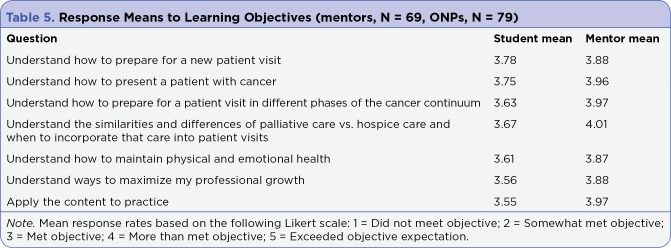
Response Means to Learning Objectives (mentors, N = 69, ONPs, N = 79)

The qualitative measure consisted of the following open-ended questions: "Identify two things you learned from this activity" and "What additional content should be included for future continuing education modules?" When asked the open-ended question "Identify two things you learned from this activity," both ONPs and mentors identified communication strategies, self-promotion, and professional development. In addition, ONPs and mentors requested additional content related to hematology. Furthermore, mentors requested more content in the areas of oncologic emergencies and symptom management to be added to the ONc-PoWER modules.

## DISCUSSION

Continuing education can enhance the professional nursing practice upon completion of a basic nursing education and provide essential evidence-based practice information in a format conducive to "on-the-job" learning. Florence Nightingale, in her Notes on Nursing, argued for the importance of ongoing education for nurses through observation, experience, new knowledge, and evidence (Nightingale, 1898). The challenge for continuing education programs is to provide educational activities that bridge the knowledge gap between formal education and professional practice for all nurses. This is particularly true for nurse practitioners in highly specialized practice settings such as oncology.

ONc-PoWER was created using a familiar web-enhanced platform. In this evaluation, the five ONc-PoWER modules were completed by 79 dyads, each comprising an ONP new to practice and his or her on-site mentor, from hospitals and clinics across the United States. A web-enhanced platform such as ONc-PoWER, which incorporates evidence-based teaching strategies (e.g., experiential learning), allows the learner to log in whenever it is convenient for his or her schedule, thus permitting the learner to proceed through the learning content at his or her own pace. Furthermore, the use of web-enhanced instruction for ONc-PoWER makes its content available to more ONPs who are new to practice, which eliminates some of the barriers presented by brick-and-mortar educational programs (e.g., commuting to a location to physically attend class). ONc-PoWER provided this flexible access necessary to provide essential cancer care knowledge to ONPs and mentors from 27 US states.

Web-enhanced learning, coupled with opportunities for local clinical application, is an ideal method for ensuring standard entry-level competency while providing a realistic, clinical orientation specific to the local culture of cancer care practices (LeFlore et al., 2011). For example, using qualitative methods, Bridgemohan, Levy, Veluz, and Knight (2005) found that participants using a technology-based learning format reported learning to be more flexible and more interactive with greater auditory and visual appeal. Similar findings were found with master’s-level nurse educators using a virtual simulation in an education certificate program (Foronda, Lippincott, & Gattamorta, 2014). Students taking part in the simulation revealed that the activity enabled them to appreciate the use of technology as a teaching tool and helped them to develop their skills for future employment (Foronda et al., 2014). In terms of graduate student preferences for web-enhanced coursework, Carlson and Jesseman (2011) report that flexible scheduling and the ability to think about responses before submitting them into course evaluation methods are advantages of designing and conducting educational programs. The 6-month time frame of ONc-PoWER provided this flexibility to the participants for module completion.

As demonstrated by this evaluation, ONc-PoWER’s five modules provided an effective opportunity for the new ONP to increase his or her knowledge base related to cancer care. Although this project was specific to ONPs, the ONc-PoWER curriculum can be easily adapted to include PAs new to cancer care. After completing ONc-PoWER, the ONPs rated their learning experience in terms of not meeting, meeting, or exceeding their expectations vis-à-vis the objectives of the modules (see Table 5). Participants’ survey results verify that the ONc-PoWER content is easy to learn, applicable to practice, and fills the gap between the curricula of standard graduate programs in nursing and specialized professional practice. The effectiveness of web-enhanced learning has been studied over the past decade, as more programs and areas of study move away from the traditional face-to-face programs in response to the accessibility needs of an increasingly diverse student body (Allen & Seaman, 2013; Bangert & Easterby, 2008). Additionally, web-based learning has documented lower attrition, increased user flexibility, and appeals to a wider audience (Horiuchi, Yaju, Koyo, Sakyo, & Nakayama, 2009). In cancer care, this is particularly important for ONPs who face barriers to continuing education in nursing and cancer care. For example, many ONPs work in low-resource areas and may be unable to attend traditional classes presented in a face-to-face format. One participant from a rural area noted that without ONc-PoWER, she would have needed to rely on a part-time, semi-retired oncologist for additional education and he was not readily accessible for cancer care training.

## CONCLUSION

A web-enhanced continuing education program that incorporates ONS entry-level competencies, adult learning theory, and principles of effective online education can be a viable option for ONPs new to professional practice who need additional training to better transition to their new role. This mode of instruction, featured in ONc-PoWER, which was further coupled with an on-site mentor, increased the ONP’s knowledge related to cancer care. Furthermore, the web-enhanced ONc-PoWER modules provided flexible access for ONPs across the United States. Nursing faculty and nurse educators should consider the use of well-designed, asynchronous web-enhanced instruction—something akin to ONc-PoWER—as a teaching strategy to promote continuing cancer care education for other specialty NPs during their first year of professional practice.
